# *De Novo* Whole-Genome Sequence of *Xylella fastidiosa* subsp. *multiplex* Strain BB01 Isolated from a Blueberry in Georgia, USA

**DOI:** 10.1128/genomeA.01598-16

**Published:** 2017-02-09

**Authors:** Christopher Van Horn, Chung-Jan Chang, Jianchi Chen

**Affiliations:** a USDA-ARS San Joaquin Valley Agricultural Sciences Center, Parlier, California, USA; b Department of Plant Pathology, University of Georgia, Griffin Campus, Griffin, Georgia, USA

## Abstract

This study reports a *de novo*-assembled draft genome sequence of *Xylella fastidiosa* subsp. *multiplex* strain BB01 causing blueberry bacterial leaf scorch in Georgia, USA. The BB01 genome is 2,517,579 bp, with a G+C content of 51.8%, 2,943 open reading frames (ORFs), and 48 RNA genes.

## GENOME ANNOUNCEMENT

*Xylella fastidiosa* is a phytopathogenic, xylem-limited, and nutritionally fastidious bacterium associated with diseases in many economically important plants (1). In 2009, by fulfilling Koch’s postulates, Chang et al. reported a strain of *X. fastidiosa* causing leaf scorch disease in blueberry in Georgia, USA, and designated the disease blueberry bacterial leaf scorch (BBLS) (2). BBLS is a growing threat to blueberry production in the southern United States, particularly to the highbush cultivars (*Vaccinium corymbosum* interspecific hybrids). Based on total sales, the blueberry is among the top fruit commodities in the state of Georgia. The public demand for efficient BBLS disease control is high, which requires knowledge of the pathogen. The nutritionally fastidious nature has been a barrier for *X. fastidiosa* research based on traditional culture-based methodologies. Many basic biology issues of BBLS bacteria remain unclear. For example, while isolation and enzyme-linked immunosorbent assay (ELISA) testing methods could detect the bacterium, they could not determine the subspecies status of the BBLS strain, which is important information for disease epidemiology (3). However, recent advancements in next-generation sequencing (NGS) technology has opened a new venue to study *X. fastidiosa* through whole-genome sequencing and analyses. To provide baseline information for further disease study, this project reports a *de novo* genome assembly of an *X. fastidiosa* BBLS strain.

Strain BB01 of *X. fastidiosa* was isolated on periwinkle wilt (PW) medium from a symptomatic blueberry (selection FL86-19) collected from Nahunta, GA. This bacterial strain was triple-cloned and cultured at 28°C for 14 days. Bacterial cells were collected by centrifugation. Total genomic DNA was extracted according to a standard procedure (4). Whole-genome sequencing was performed on the Illumina MiSeq platform (Illumina, Inc., San Diego, CA) with 250-bp paired-end reads. A total of 3.65 × 10^9^ bp of sequences was generated. Sequence reads were interleaved and assembled *de novo* using CLC Genomics Workbench (version 7.5). The BB01 genome (1,271× coverage) had a G+C content of 51.8%, with 2,517,579 bp distributed among 84 contigs ranging in size from 1,006 bp to 197,455 bp. Annotation was performed by the RAST server (http://rast.nmpdr.org/) (5). The BB01 genome was predicted to have a total of 2,943 open reading frames (ORFs) and 48 RNA genes.

To evaluate the subspecies status of BB01 strain, the sequences of the 16S rRNA gene and *nrdA* (ribonucleotide reductase subunit A) were retrieved and compared to all available whole-genome sequences of *X. fastidiosa*, including *X. fastidiosa* subsp. *fastidiosa*, *X. fastidiosa* subsp. *multiplex*, and *X. fastidiosa* subsp. *pauca* in GenBank (version 216) through BLASTn analysis. In all cases, strain BB01 clustered with members *X. fastdiosa* subsp. *multiplex*, such as strain M12 (6), indicating that BB01 is a strain of *X. fastidiosa* subsp. *multiplex*.

### Accession number(s).

This whole-genome shotgun project has been deposited in DDBJ/EMBL/GenBank under the accession no. MPAZ00000000. The version described in this paper is the first version, MPAZ01000000.
